# Dromedary camel’s welfare: literature from 1980 to 2023 with a text mining and topic analysis approach

**DOI:** 10.3389/fvets.2023.1277512

**Published:** 2023-11-09

**Authors:** Naod T. Masebo, Martina Zappaterra, Martina Felici, Beatrice Benedetti, Barbara Padalino

**Affiliations:** ^1^Department of Agricultural and Food Sciences, University of Bologna, Bologna, Italy; ^2^School of Veterinary Medicine, Wolaita Sodo University, Wolaita Sodo, Ethiopia

**Keywords:** camelids, husbandry, production, machine learning, research

## Abstract

Dromedary camels are the preferable livestock species in the arid and semi-arid regions of the world. Most of the world’s camel populations are managed under a subsistence/extensive system maintained by migratory pastoralists but intensification is getting more frequent. Even though recently the welfare of camels has been receiving more attention, in many countries there are no regulations to protect their health and welfare. The objectives of this article were to explore the main research topics related to camel welfare, their distribution over time and to highlight research gaps. A literature search was performed to identify records published in English from January 1980 to March 2023 on Dromedary camel welfare via Scopus^®^, using “Camel welfare,” “Camel behaviour,” “She-camel” and “Camel management” as search words. A total of 234 records were retained for analysis after automatic and manual screening procedures. Descriptive statistics, text mining (TM) and topic analysis (TA) were performed. The result shows that even though there were fluctuations between years, records on camel welfare have increased exponentially over time. Asia was the region where most of the corresponding authors were located. The first five most frequent words were, “milk,” “calv,” “behaviour,” “femal,” and “breed,” the least frequent word was “stabl.” TA resulted in the five most relevant topics dealing with “Calf management and milk production,” “Camel health and management system,” “Female and male reproduction,” “Camel behaviour and feeding,” and “Camel welfare.” The topics that contained the oldest records were “female and male reproduction” and “camel health and management system” (in 1980 and 1983, respectively), while the topic named “camel behaviour and feeding” had the first article published in 2000. Overall, even though topics related to camel behaviour and welfare are receiving more attention from academia, research is still needed to fully understand how to safeguard welfare in Dromedary camels.

## 1. Introduction

The domestication of Camels started around 3,000 B.C. in South-East Arabia and South-West Central Asia (1, 2). The genus *Camelus* contains three species, the one-humped camels or Dromedary (*Camelus dromedarius*), the two-humped camels or Bactrian (*Camelus bactrianus*) (1) and the recently identified, never domesticated two-humped *Camelus ferus* located in the Mongolian Great Gobi, in the Chinese Lop Nur, Taklamakan deserts (3). Usually, the Bactrian inhabits the northern colder areas and Dromedary is found in southern hotter areas of the old world. Dromedary camels (*Camelus dromedarius*) are found in different African and Asian countries (1) where they have primary economic, social, and cultural values (4). Dromedary camels are the main livestock species reared in the arid and semi-arid regions of the world where other livestock could not survive; their biological and physiological particularities enable them to withstand days in harsh environments with water and feed shortage (2, 5).

The world camel population is increasing continuously. In 2021, Chad, Somalia, and Sudan were the three countries with the largest camel populations, with 9.4, 7.4, and 4.9 million camels, respectively; it is estimated that the world camel population could reach 60 million in the next 25 years (6, 7). In the majority of nations, camel production is still a subsistence/extensive system, mostly maintained by migratory pastoralists in arid and semi-arid regions (8). Dromedary camels are multi-purpose livestock, used for carrying goods, in agriculture (ploughing and cultivation), as drought animals, for transportation and as a source of food (milk and meat) (1, 3, 8). In addition, in Middle Eastern countries, Dromedary camels are kept for sporting activities, such as camel racing, and for beauty contests (3). In recent years, there has been an increase in intensive camel production in peri-urban farms, supplying milk to urban dwellers (9). The growing intensification of camel husbandry systems is determined by the increase in demand for camel milk due to its nutritional and health enhancement benefits (10). The trend towards intensification in camel husbandry is also expected to increase in the coming years due to various reasons, including climate change (11). As a result of global warming, the temperature of the environment is increasing resulting in desertification, drought, and food shortages. Due to their adaptability and sustainability in extremely arid environments, Dromedary camels are therefore viewed with increasing interest even by countries where this livestock species was not traditionally bred (9). As interest in this animal species grew, so did the number of scientific works aimed at investigating its physiology (12), genetics (13), and welfare (14–16).

Animal welfare science has advanced rapidly in the last 30 years as a result of increased understanding of animal motivation, cognition, and the complexities of social behaviour (17). The methods employed in animal breeding, transportation and killing are subjects of public interest that lead to debates and activism (18). Meeting the rising demand for animal products without ignoring societal issues requires improving the efficiency of current animal production systems (19). Good welfare requires disease prevention, appropriate veterinary care, shelter, management and nutrition, a stimulating and safe environment, humane handling, and humane slaughter or killing of animals (20). There are various reasons for the growing demand for animal welfare enhancement, which is recognised globally through enaction of policies and regulations (21). Even though the attention given to welfare issues of Dromedary camels has increased in recent years (3) there are still no regulations establishing minimum requirements to protect the health and welfare of Dromedary camels (22, 23).

The concept of animal welfare has also changed a lot in the scientific field. Starting with the discussion of ethical positions, the concept of animal welfare has evolved (17), seeking a balance between public perception, and concepts of production, health, and psycho-physical well-being of animals. The term “animal welfare” can therefore be approached from different points of view and applied to different areas of study that are increasingly multidisciplinary. The identification of the topics most associated with animal welfare terms and their temporal changes provides a picture of this evolutionary process and suggests present and future trends. Bibliometrics analyses applied to literature allow for the screening of a vast number of records at both macroscopic and microscopic levels (24, 25). Text mining (TM) and topic analysis (TA) are extensions of classical bibliometric analyses and are machine learning-based techniques. These techniques are useful to investigate the trends in the scientific literature (26–28). By utilizing TM, it is possible to classify and group textual information, enabling the generation of outcomes like word frequency distribution, pattern identification, and predictive analytics that are not easily attainable with standard data analysis methods (29).

Therefore, this systematic review aims to evaluate literature dealing with Dromedary camel welfare that was written from January 1980 to March 2023 using TM and TA methods. This review was intended to improve understanding of topics associated with welfare of Dromedary camels, following their evolution through time and countries of publication, and to detect any gaps in knowledge and need for future research.

## 2. Methodology

### 2.1. Literature search

**Figure 1:**
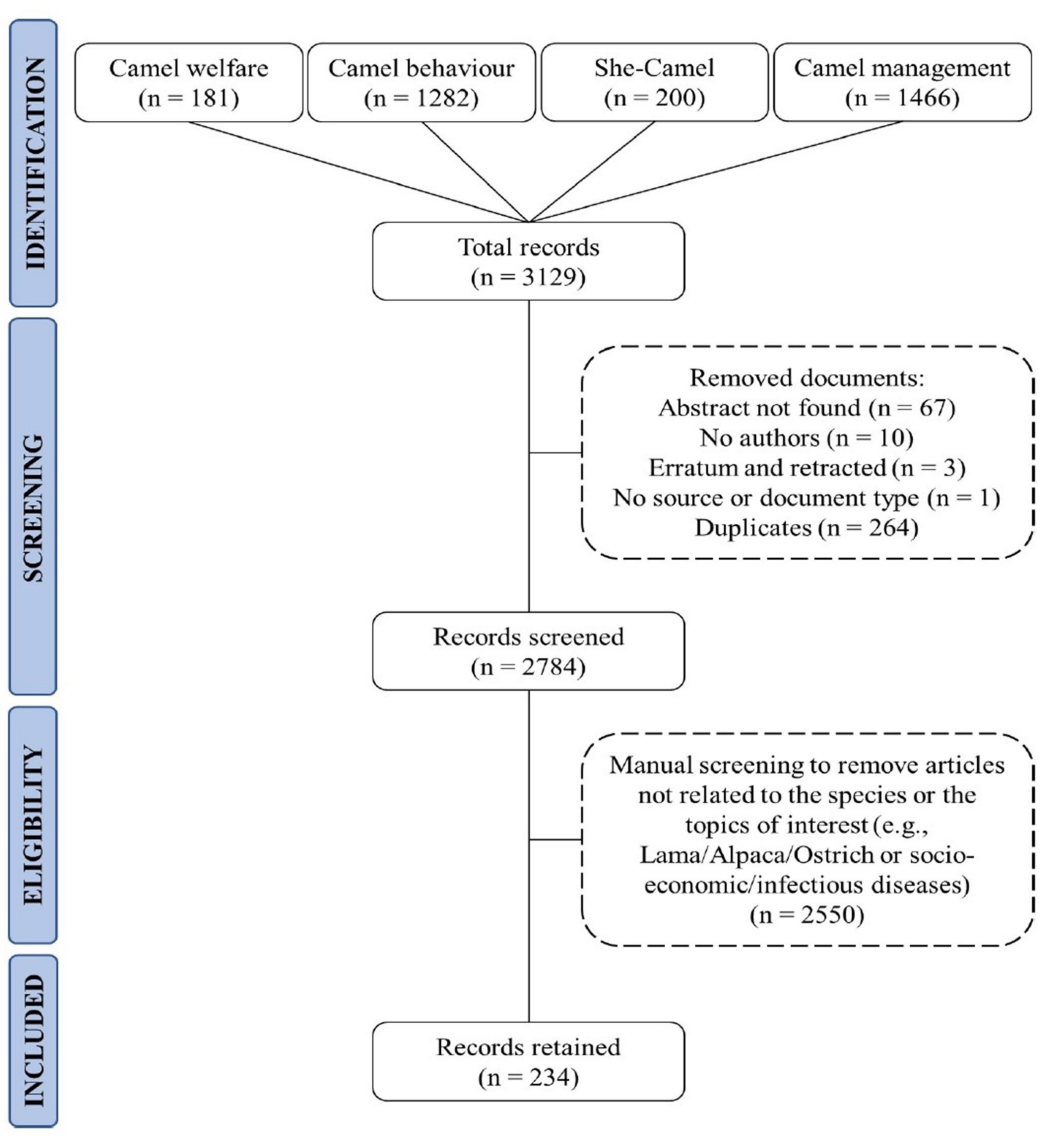
Flow chart of the scientific literature showing the number of records discovered with each keyword sequence and the total number of records included in the review of camel welfare literature.

A systematic scientific literature search about Dromedary camel’s welfare was performed to identify English records using Scopus^®^ (i.e., the abstract and citation database for Elsevier^®^). The search was conducted on the 21st of March 2023. The keywords that were used for the search were included: “Camel welfare,” “Camel behaviour,” “She-camel” and “Camel management.” Veterinary, biochemistry, genetics and molecular biology, social sciences, immunology, microbiology, multidisciplinary, neuroscience and engineering were included as the subject areas in the search. A Microsoft spreadsheet (Microsoft Excel^®^, v16.0, Redmond, WA, United States), which included all the records published from 1980 to the day of the search, was downloaded from Scopus^®^. In the spreadsheet, each line reported a record and each column the information extracted from the record such as: year of publication, authors, abstract, affiliation, country, regions, record type (e.g., article or review) and the source of publication (e.g., Journal title). The records were then screened and those that had no abstract, no author name, retracted or erratum, no source, or duplicates were excluded automatically. Finally, manual screening was performed by the researcher (MF) based on the topic and the species discussed in each record to decide the eligibility of the record for inclusion in the final analysis. In particular, records related to other species (e.g., Lama, Alpaca, ostriches) and other topics (e.g., socio-economic, infectious disease) were excluded. Records that studied Dromedary and/or Bactrian camels in combination with other livestock (e.g., buffaloes, cows, goats etc.) were retained. Records that were difficult to categorise were checked by a welfare expert (BP), who made the final decision on whether they should be excluded or included in the study. The screening process is further summarised in a flow chart indicating all the steps with the number of records excluded or retained in each step (Figure 1). Descriptive statistics based on the regions of origin of the records, countries, and year of publication were performed using Excel Pivot tables and results are presented as graphs. The regions of origin of the records were identified based on the affiliation of the corresponding author and, if not indicated, of the first author.

### 2.2. Text mining

An additional Excel sheet was prepared containing two columns namely “ID” and “abstract” of the records for TM analysis. The authors standardised the corpus of records using only British English, as some words in the corpus were spelt both in American and British English. In particular, the handling process was performed on the word pairs “behaviour”-“behavior,” “analyse”-“analyze,” “program”-“programme.” Therefore, TM analysis was performed on the abstract of the records that were retained for the final analysis (26). The corpus of records was submitted to pre-processing steps according to Sebastiani (30). In detail, the text was reduced to lowercase, and unusual symbols (e.g., “@,” “/” or “*”), punctuations, numbers, and English stop words (e.g., “the,” “a,” “and,” “on,” “at”) were removed. In addition, researchers removed words strictly associated with the search or commonly used in scientific articles, namely “camel,” “camels,” “group,” “groups,” “test,” “time,” “significantly,” “significant,” “significative,” “significance,” “study,” “studies,” “she,” “animal,” “animals,” and “management.” At the end of these processes, the extra spaces within words were removed. Text tokenization was performed to reduce words to their root. The next step was to create a document-term matrix (i.e., a matrix that contains the records along the rows and the terms along the columns) as reported in the literature (26). In order to identify the weight of each word, a term frequency-inverse document frequency technique (TFIDF) was applied (31). This is the frequency of a term adjusted for how extensively it is used, demonstrating the importance of a word in the overall collection of records (27). In this study, as reported in the literature (27), the first set of 25 words was presented as a histogram. In our corpus of records, to obtain these 25 words, the TFIDF cut-off was set to 1.96, which represented the weight of the 25th word. A cloud of the most relevant words (TFIDF ≥1.96) was also constructed using the website,^1^ with larger character sizes indicating a higher TFIDF value. Associations among the most relevant words (TFIDF ≥1.96) and all the record terms were identified, using a grade of correlation ≥0.3. To calculate associations, the frequency with which two words emerge together was considered. Particularly, if two words always emerge together the association is 1 and if they never emerge together the association is −1. The TM analysis was carried out in R environment (32) using functions from the package’s “tm,” “SnowballC,” “ggplot2,” “dplyr,” and “tidyverse.”

### 2.3. Topic analysis

In order to perform TA, Latent Dirichlet Allocation (LDA) approach was applied (33). LDA is a hierarchical Bayesian technique that learns a set of theme topics from words that appear together frequently in records. A single subject can be thought of as a multinomial distribution of words, and a single record as a multinomial distribution of latent topics. The model infers the hidden topic structure from the observed records and words, generating per-record topic distributions and per-topic word distributions (33). LDA function with Gibbs sampling option of the “*topic models”* package in R was used (34), and the R library “*tidytext”* was used to present the graphic of the commonest words of each topic and their relative probability to belong to that topic (beta value). Before TA commenced, the number of topics in which the corpus had to be split was determined. However, because the “ideal” number is generally unknown, trials with 5, 6 and 8 topics were performed and the most suggestive panel among them was chosen based on consensus among the researchers. Once the definitive number of topics (*n* = 5) was identified, each researcher independently named them providing an indicative label. The final label of each topic was discussed and defined with the agreement of all researchers. To classify the topics, the cumulative probabilities (cp) of the first 20 words of each topic were calculated. Topics were shown according to this classification (i.e., topic 1 has the highest cp).

## 3. Results

### 3.1. Descriptive statistics

Out of 3,129 abstracts that were downloaded from Scopus^®^, 234 (7.45%) fulfilled the screening and eligibility criteria and were retained. Not pertinent [i.e., about other species, other topics such as socio-economic topics etc. (81.49%)] was one of the main reasons to exclude records from further analysis. The other most frequent reasons for exclusions were the following: duplicates (8.44%), no abstract (2.14%) and no author found (0.32%) (Figure 1). The type of records retained were research articles (205/234; 87.6%), reviews (14/234; 5.98%), book chapters (9/234; 3.85%), conference papers (3/234; 1.28%), notes (2/234; 0.85%) and books (1/234; 0.43%).

**Figure 2:**
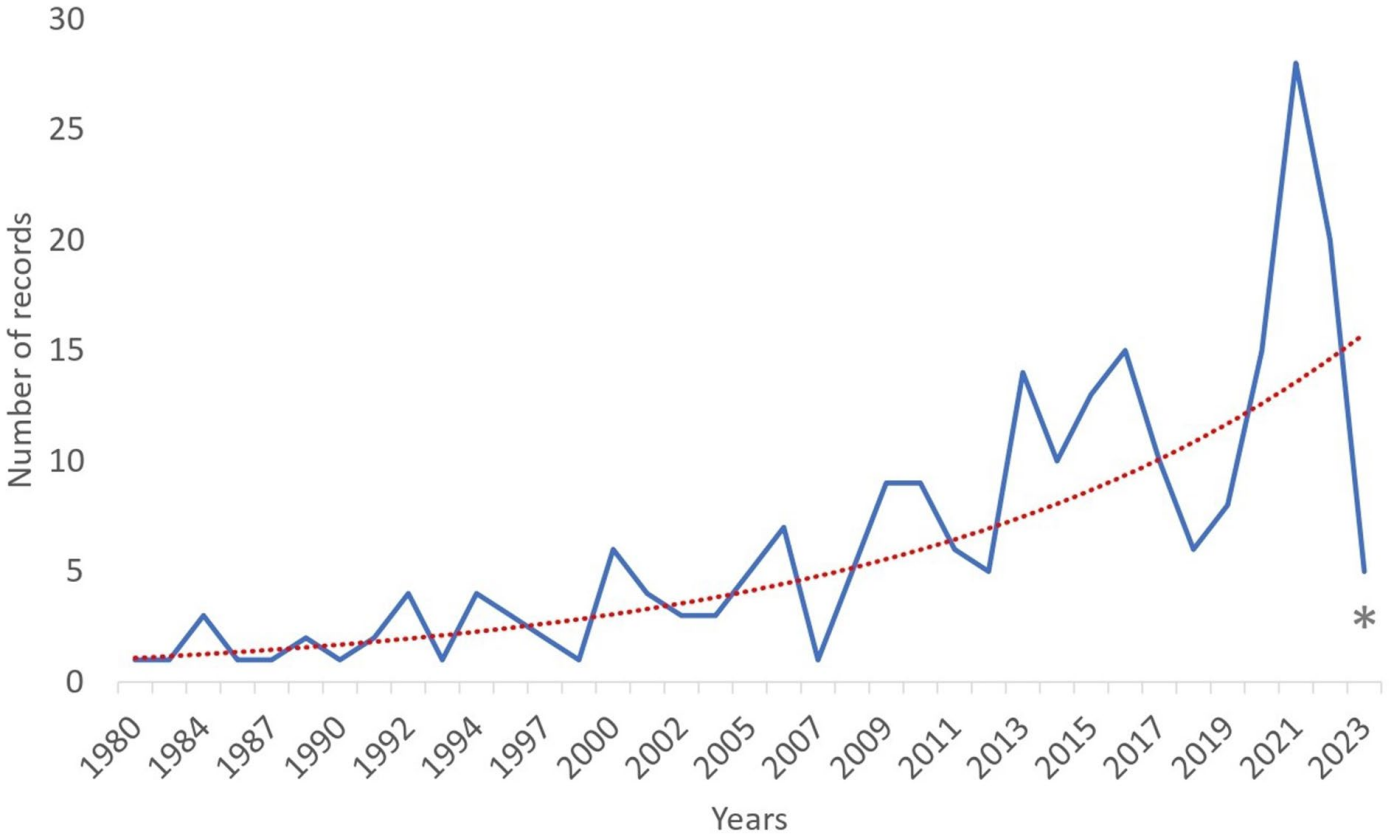
Number of records distributed by publication year (1980–2023) of 234 records selected for inclusion in the review. The exponential trend is represented by the dashed red line. * Indicates that results in this year are related to the period from January to March.

**Figure 3:**
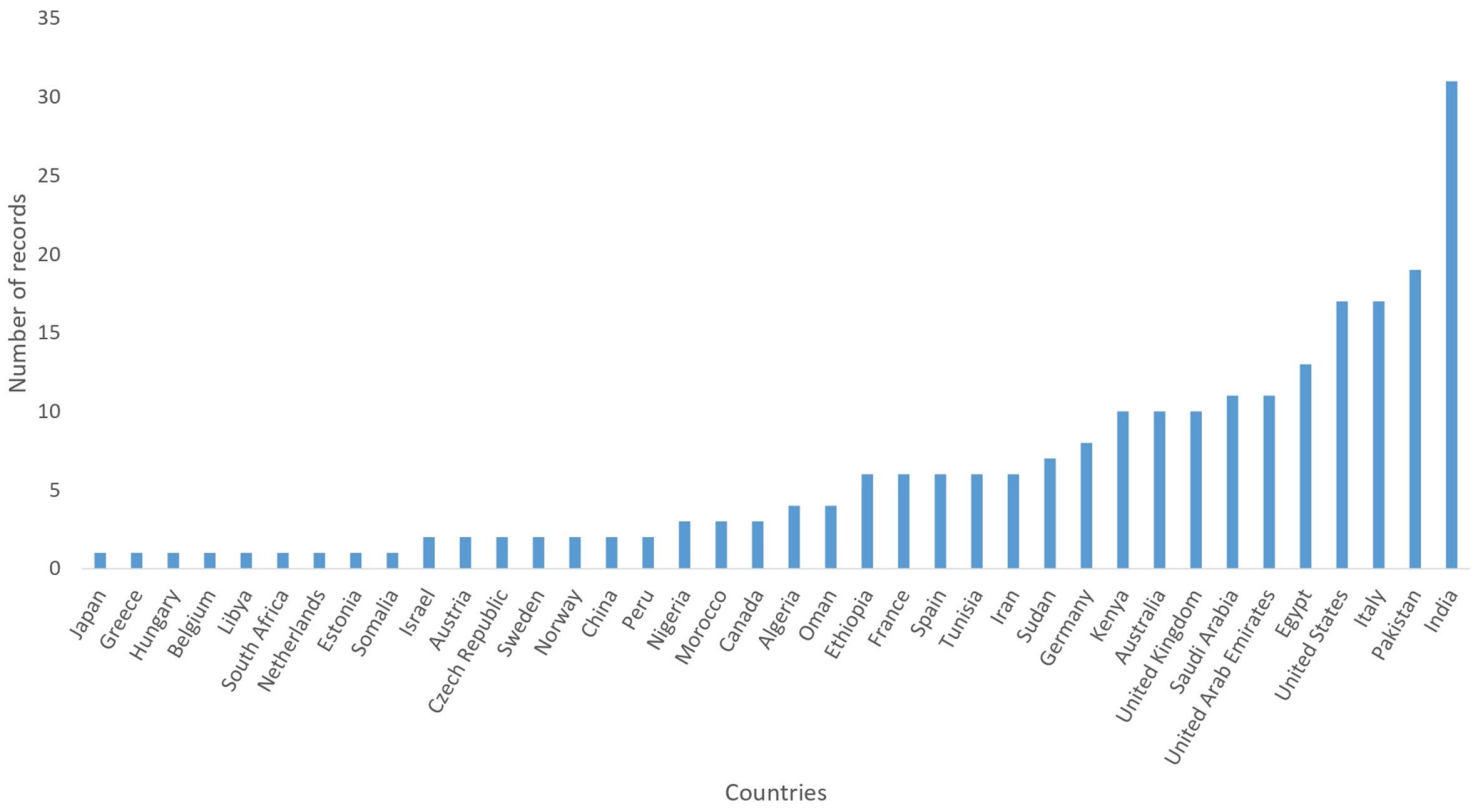
Number of records based on countries of the 234 records selected for inclusion in the review. The countries are based on the nationality of the corresponding authors.

**Figure 4:**
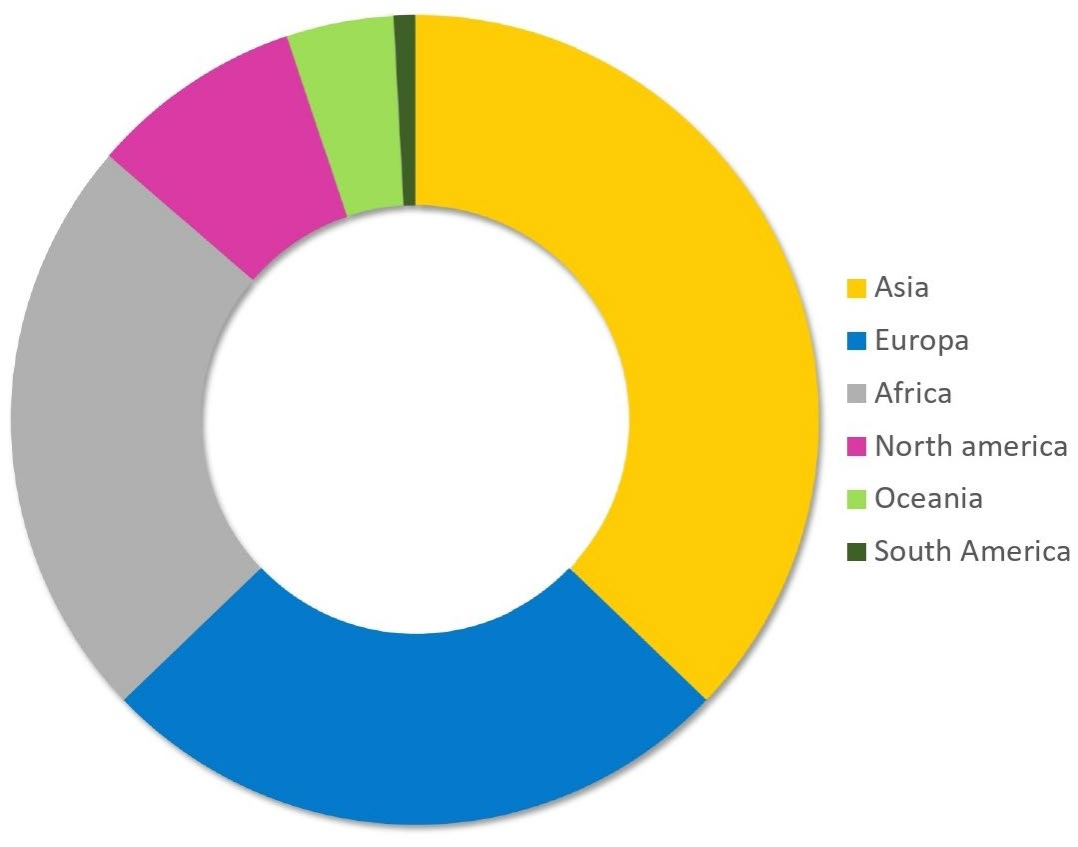
Distribution of records based on regions. The regions were determined based on the nationality of the corresponding authors.

The total number of records published per year has increased exponentially over time (Figure 2). Based on the corresponding author address, India (31/234; 13.25%), Pakistan (19/234; 8.12%), United States of America (17/234; 7.26%), Italy (17/234; 7.26%) and Egypt (13/234; 5.56%) were the countries from which most articles were submitted (Figure 3). Asia (37.17%) was the region where most of the corresponding authors were based followed by Europe (25.64%) and Africa (23.5%) (Figure 4). The records were published in 87 different scientific journals (Supplementary material S1).

### 3.2. Text mining

**Figure 5:**
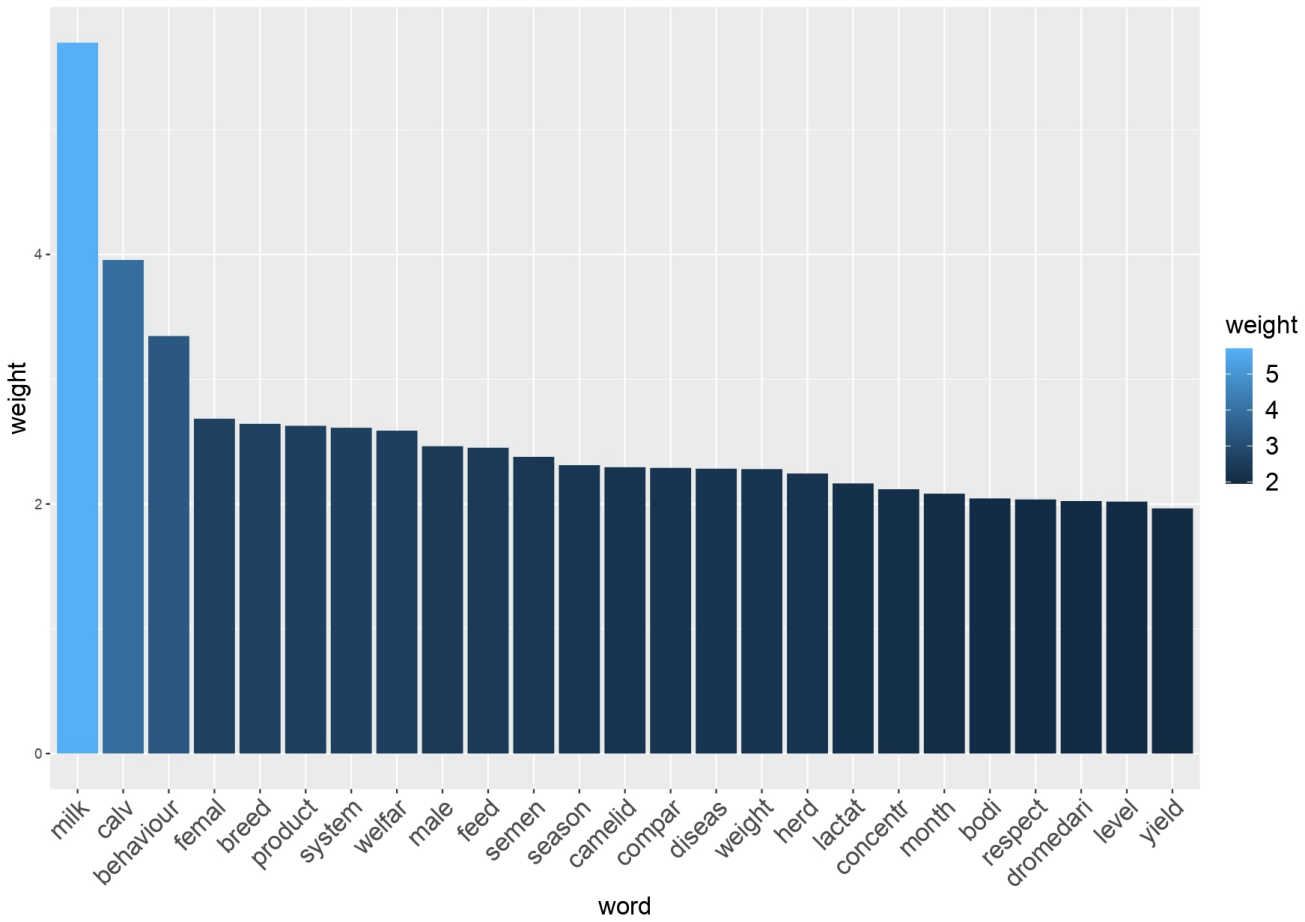
Histogram showing the most relevant words (TFIDF ≥1.96) of 234 records selected for inclusion in the study and their respective weights.

**Figure 6:**
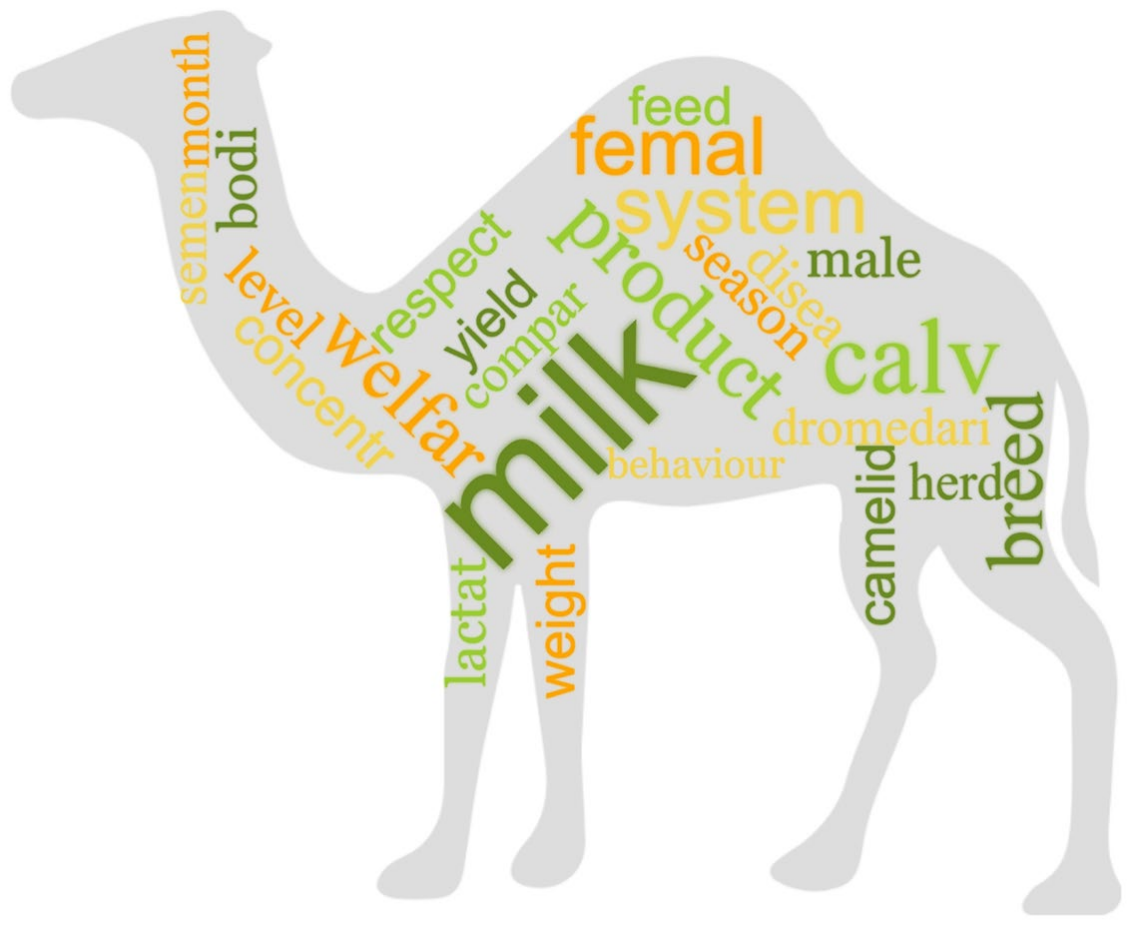
Word cloud representing the most relevant words of the corpus of 234 records selected for inclusion in the review. The size of the words is proportional to the weight they have in the corpus.

After pre-processing of the data and reduction of sparseness (i.e., exclusion of the “rare words”), 1,346 terms were retained from the selected 234 records. The most relevant words (TFIDF ≥1.96), according to the TFIDF ponderation system, are represented in a histogram (Figure 5) and a word cloud (Figure 6), with the font size proportional to the TFIDF of each word. The words with the highest TFIDF were “milk” (5.71), followed by “calv” (3.97), “behaviour” (3.37), “femal” (2.70), “breed” (2.65), “product” (2.63), “system” (2.62), “welfar” (2.59), “male” (2.47) and “feed” (2.46). The word with the lowest TFIDF was “stabl” (0.1). The associations between the most relevant words (TFIDF ≥1.96) and the other words of the matrix are shown in Table 1. The words “female,” “Breed,” “Feed,” “Season,” “Concentr” and “Level” showed no significant correlation (with correlation grade ≥ 0.3) with other words.

**Table 1:** Associations between the most relevant words (TFIDF ≥1.96) and the other words present in the corpus of 234 records selected for inclusion in this review.

Words (TFIDF ≥ 1.96)	Words associated (grade of correlation ≥ 0.3)
Milk	Udder (0.55); machin (0.53); letdown (0.45)
Calv	Interv (0.52); first (0.51); februari (0.42); open (0.41)
Behaviour	Sexual (0.68); heat (0.63); induc (0.57); habitat (0.55); specif (0.47); adapt (0.44); natur (0.40)
Product	Meat (0.42)
System	Semi-inten (0.64); Khartoum (0.40)
Welfar	Buffalo (0.4)
Male	Intromiss (0.44); mount (0.41)
Semen	Collect (0.73); artifici (0.62); sperm (0.61); ejacul (0.53); insemin (0.46); preserv (0.44); modif (0.43); resili (0.42)
Camelid	World (0.59); american (0.56); south (0.51); suscept (0.49); metabol (0.45); chapter (0.44)
Compar	Quit (0.69); allot (0.66); biometr (0.61); khejri (0.58); prosopi (0.58); less (0.57); iron (0.55); zinc (0.55); cost (0.53); trial (0.53); manger (0.49); copper (0.48); inten (0.48); wither (0.46); hind (0.45); random (0.45); total (0.43); gain (0.41); economy (0.40)
Disea	Origin (0.55); scope (0.48); introduc (0.47); togeth (0.46); difficulti (0.45); interpret (0.45); infecti (0.42); worm (0.41)
Weight	Gain (0.58); birth (0.52); growth (0.45)
Herd	Mortal (0.42)
Lactat	Fourth (0.63); highest (0.56); pariti (0.55); composit (0.48); peak (0.47); similar (0.46)
Month	Januari (0.42); februari (0.40)
Bodi	Circumf (0.50); quit (0.47); allot (0.44); trial (0.41)
Respect:	Eight (0.47); parturit (0.47); symptom (0.45); newborn (0.42)
Dromedari	Recent (0.43)
Yield	Highest (0.58); composit (0.56); pariti (0.56); peak (0.47); record (0.45); persist (0.44); similar (0.41)

The correlation grade is written between the brackets. The grade of correlation was set at ≥0.3.

### 3.3. Topic analysis

**Figure 7:**
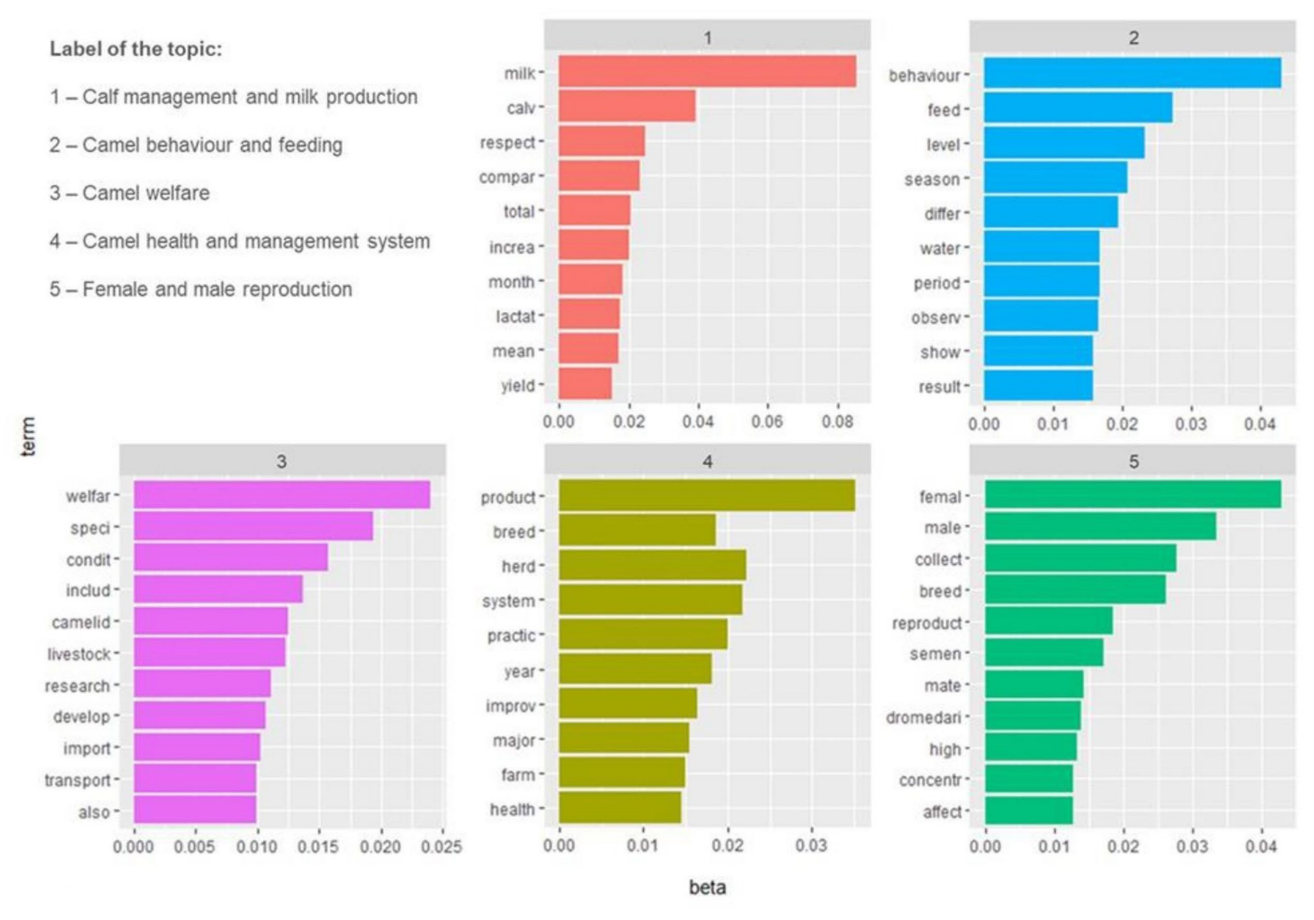
Histograms showing the ten most frequent words within the five topics revealed with LDA analysis of 234 records selected for inclusion in the review. Beta indicates the relative probability of each term belonging to that topic. The topics were ordered from 1 to 5 in accordance with their cumulative probabilities.

**Figure 8:**
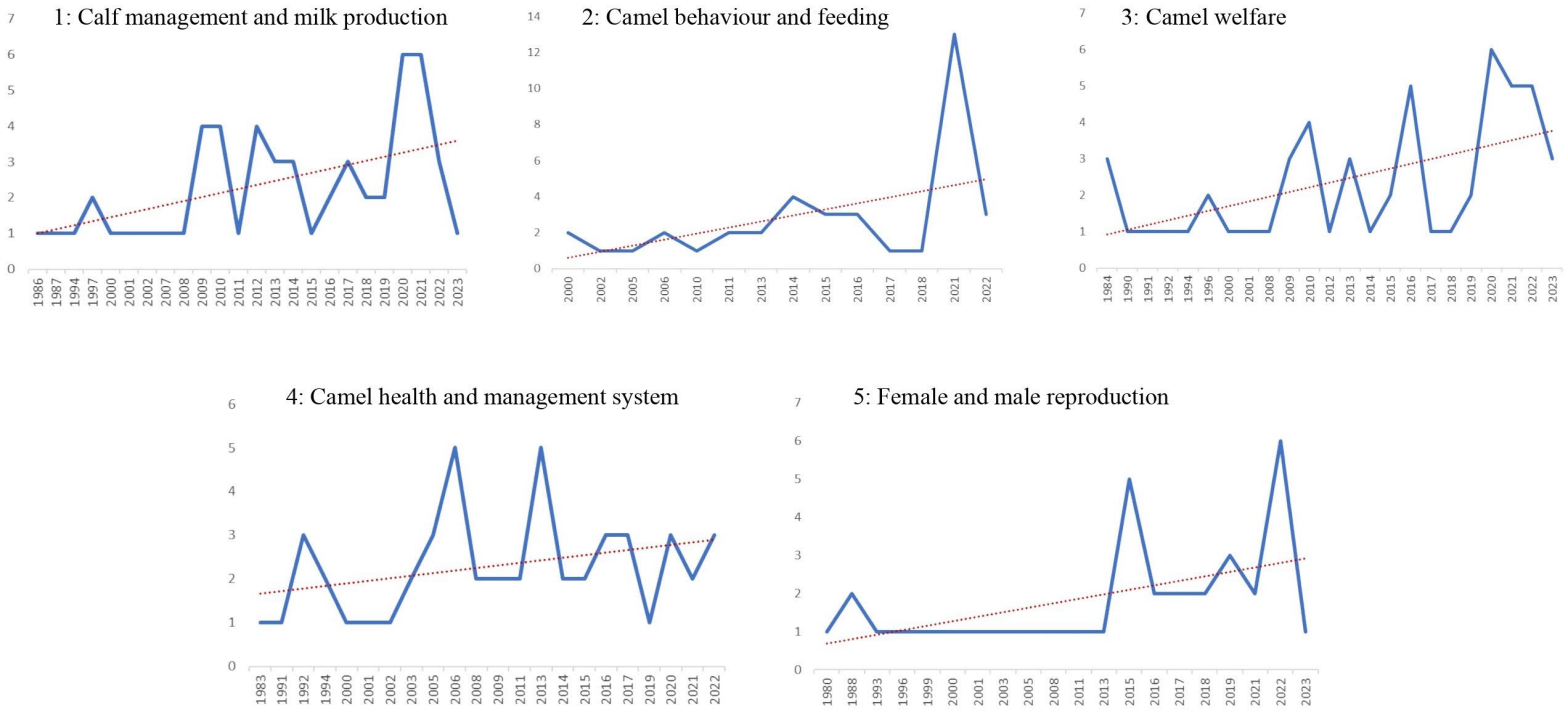
Number of records included in each topic starting from the year of the first publication. The results for 2023 are for January through March.

Five topics were chosen as the ideal topics and labels were assigned to each of them. The name of each topic as well as the number of records contained in each topic are shown in Table 2. Figure 7 shows the topics numbered from 1 to 5 according to the cumulative probabilities (cp), and the first 10 words for each topic. The topics containing the oldest records were those named “female and male reproduction” and “camel health and management system” (in 1980 and 1983, respectively), while the topic named “camel behaviour and feeding” were contained the most recent records, published in 2000 (Figure 8). The TA performed with 6 and 8 *a-priori* numbers of topics are shown in the Supplementary material S2.

**Table 2:** Numbers and labels of the 5 topics revealed with LDA analysis of 234 records selected for inclusion in the review and number of records included in each topic.

Number of the topics	Label of the topic	Number of records per topic
1	Calf management and milk production	55
2	Camel behaviour and feeding	39
3	Camel welfare	54
4	Camel health and management system	50
5	Female and male reproduction	36

The final labels of the topics have been chosen with the agreement of all researchers.

## 4. Discussion

Performing a literature review is a crucial approach to analyse the present status of a specific topic and offer guidance for future research directions (33). This systematic review performed using the statistical methods of TM and TA yielded valuable information about the welfare of Dromedary camels from a vast collection of scientific literature written over the last four decades. These techniques enable the authors to evaluate diverse themes in the subject area and identify gaps in knowledge.

The number of records on Dromedary camel welfare has increased exponentially over the years. This was expected, because animal welfare research, as an interdisciplinary field of research that started to develop in the 1970s, has gained prominence since then. The driving force has been public concern about the welfare of animals kept in different husbandry systems (35). Additionally, animal welfare and social and environmental sustainability are also becoming more and more significant (19). Therefore, the increasing concern for animal welfare and a growing belief that farm animal welfare should be protected and improved (36), coupled with the recognition of the unique challenges faced by Dromedary camels, has driven a rapid rise in the number of records addressing their welfare (3). Findings reported here show that there is a high number of records, and the number has steadily been increasing particularly from 2020. According to recent bibliometric research by Kandeel et al. (37), the year 2020 marked a highly productive year for camel research. The authors suggested that the recent surge in camel studies could be attributed to the availability of an increased number of records and special issues specifically focusing on camels and their role as natural reservoir species for respiratory virus outbreaks, such as MERS-CoV infection. Furthermore, this remarkable increase in records has also been driven by recent international projects and collaborations, such as the *CA.*RA.VA.N network (towards a CAmel tRAnsnational VAlue chaiN; https://anr.fr/Project-ANR-16-ARM2-0002) and the International Camel Consortium for Genomic Improvement and Conservation^2^ running in those years.

Most of the records on camels came from Asia; this is not surprising considering that Asia has the second-largest Dromedary camel population globally (6). Furthermore, the intensification of Dromedary camel production has been on the increase in Asian countries (9–11), and these nations boast dedicated research centers focused on camel research (38). Similarly, Iglesias Pastrana et al. (39) in their bibliometric research about camels indicated that countries with traditionally well-established camel farming are responsible for the papers with the highest academic impact. However, in our findings, Europe emerged as the second-leading region in terms of published research, despite having a relatively small population of Dromedary camels. This achievement can be credited to collaborative research conducted between European researchers and experts from traditional camel-rearing countries (38). Several European countries, such as France, Germany, Spain and Italy, have often been involved as partners in research projects with Africa and Asia. International research projects and collaborations on camels have largely benefited from the inclusion of research teams from African and Middle Eastern countries with well-established traditional camel breeding and production systems as partners (39). However, researchers working in more advanced countries or research centers with a long history of recognised scientific expertise often play important roles in coordinating or directing these international projects, which may explain why, based on the address of the corresponding authors, Europe is the second geographical region publishing on camel welfare (39). It is also possible that the significant funding support provided for camel research by the European Commission (EC) (37) could have contribute to increased scientific interest towards this livestock species. Finally, the growing interest in camels shown by various Western countries, such as Italy, can also be explained by the interest raised by this species from a climate change perspective. Dromedary camels are seen as one of the most sustainable livestock species due to their ability to produce even in arid and extreme environments (3, 11); this feature is seen with increasing interest from Mediterranean countries, where summers are increasingly arid (40).

Despite the impact of climate change on water and food resources, the world demand for animal sources products is rising, particularly in developing nations (41). As a result, the demand for camel and goat milk is estimated to triple by 2050 in different African regions (42). Achieving adequate animal welfare might be crucial for increasing the production and safety of animal products to satisfy the demands of the consumer (19). It is therefore not surprising that the first five most frequent words with the highest TFIDF were “milk,” “calve,” “behaviour,” “female,” and “breed.” Camel milk acceptance and commercialization have increased over the years, and it is being used as treatment for chronic disease conditions like diabetes and peptic ulcers (43). Dromedary camel milk is similar to human milk, and its lower-calorie content makes it ideal for persons with diabetes or obesity (23). Nowadays the Dromedary camel milk market has increased, making camel production more specialised in dairies, and leading to the advancement of camel milk production (3). The occurrence of “calve” as the second most frequent word was as expected, given that most of the scientific literature addresses dairy camels, and as camel dairy farms become more intensive, calves are moved away from their mothers (11). Much attention is therefore needed in the management practices of calves, prioritizing the identification of management strategies for the improvement of calf health and welfare. From the articles retained and analysed it is evident that a lot of attention has been placed on the growth performance and welfare of calves in different camel management systems, such as semi-intensive and traditional camel husbandry systems (44, 45). “Behaviour” was also a term frequently associated with camel welfare. In general, animal behaviour is a highly frequent topic of investigation in animal welfare. Researchers examine behaviour under various conditions to determine behavioural patterns and responses. Similarly, in camels, the retrieved studies explored how camels behave in different housing setups and environments, during husbandry and reproduction, and while feeding. The purpose is to evaluate the welfare of camels and gain insights into how their behaviour changes under different circumstances and how behaviour can be used to assess their welfare condition (16, 46–48). As with other animals, camelids do have behavioural needs that must be met to ensure their welfare. These include the possibility to express species-specific behaviours, prevent illnesses, and live in a suitable social setting (23). Overall, the TM analysis picked the most frequent words associated with Dromedary husbandry, management, milk production, calf management and welfare.

This review highlighted the prominence of welfare-related studies in dairy camels. According to the cp statistical analysis of the topics, the most important was “Calf management and milk production” (topic 1). The articles selected for inclusion in the analysis reflect a strong scientific emphasis on calf management and the enhancement of calf welfare in different camel husbandry systems through the evaluation of behavioural and physiological indicators, with the objectives of producing camel milk without affecting calve performance, health, and welfare. Additionally, camels are social, calm, and peaceful herd animals with close bonds among themselves and with their offspring. Physical and visual contact with the calf is essential for milk production to continue (49), and this could explain the frequent occurrence of the words “milk” and “calv” in the LDA analysis. Until recently, despite camels being a significant food source in arid and semiarid regions, their milk production potential has not been exploited. Dromedary camels have long lactation periods and can produce milk even in times of feed shortage, making this animal important for attaining food security and a source of income (22). The lactation length in camels typically spans around 12 months, but it can vary, ranging from 9 to 18 months (50). Milk production is influenced by a variety of factors, predominantly encompassing genetics, age, parity, lactation stage, nutrition, management, calving month, and day length (51, 52). Nonetheless, the specific impact of these elements on camel milk production remains inadequately explored, and our comprehension of their physiological processes in this context is limited (53). Historically, camel milk was solely obtained through manual milking practices within traditional, extensive, or semi-intensive farming systems. The milk was primarily consumed locally, with limited processing, and only a small portion of the production made its way to urban markets (11). However, under favourable circumstances, intensive production is performed and can present several benefits. It facilitates the efficient and economical production of quality grade raw camel milk, well-suited for subsequent processing, meeting the discerning quality demands of modern consumers. Simultaneously, this approach ensures compliance with the camels’ health and welfare needs, adhering to national and international guidelines, statutory requirements, and industry standards (11). Therefore, with the surge in global demand for camel’s milk and the consequent shift towards modern, industrial camel milk production (3), research interest in camel milk and production has increased, this may be one of the factors making this the first area of research.

The second most important topic was “Camel behaviour and feeding” (topic 2). This observation demonstrates the broad scope of behaviour-related topics in camel research, encompassing areas such as feeding behaviour, seasonal behaviour in relation to reproduction, and welfare studies. In recent times, the husbandry practices for Dromedary camels have been transitioning towards a semi-intensive system. This shift is influenced by changes in the animal’s role and the settlement of nomadic populations. However, this move towards captivity can potentially lead to limitations in the expression of various behavioural needs, impacting the camels’ social activities and leading to the manifestation of stereotypic behaviours (47). Animal behaviour is strongly influenced by the surrounding environment, and behavioural modifications serve as valuable tools for assessing the effects of different management approaches on animal welfare. Although they share many characteristics with ruminants, these animals are taxonomically, anatomically, physiologically, and behaviourally distinct, meaning that they have separate needs (23). Behaviour, health, pathology, productivity, and animal welfare are intricately interconnected. Therefore, behavioural problems serve as vital indicators of compromised welfare in these animals (14).

Until recently, the welfare of camels has not been prioritised (11). However, interest in this topic has increased enormously, so much so that the third statistically (cp) most important topic identified through LDA analysis was “Camel welfare” (topic 3). Although scientific interest in animal welfare has grown significantly as a result of consumer concern worldwide, it is still disregarded in some species, such as farmed camels. To maintain ethically acceptable conditions in these animals while they are reared, evidence-based parameters evaluating environmental and animal-based welfare indicators and scores must be established (21, 39). Animal welfare studies can provide information on the circumstances that might promote excellent welfare (54). The currently available protocols have been developed for intensive, more or less industrial, systems in developed countries. However, the principles of Welfare Quality^®^ can be used to identify animal welfare issues and risks in all systems (55). A recently published protocol for the assessment of Dromedary camel in intensive and semi-intensive systems (14, 15) adapted Welfare Quality and AWIN protocols to this species. However, the latter protocol is not useful in extensive, pasture-based systems and small, traditional farms in developing countries because of the different characteristics of the production units (19), and needs, therefore, future adaptation and validations. Moreover, improving animal welfare means ensuring that the animal experience is as positive as possible, which often requires changes in the infrastructure and practices of those responsible for the care and handling of animals (56). So, much more work is needed to understand how to measure welfare, and in particular, positive welfare, in Dromedary camels.

A crucial aspect related to animal welfare is animal health, as highlighted by the fourth most important topic identified in this review (i.e., topic 4, named “Camel health and management system”). Animal health and animal welfare are complementary but not synonymous. Without good health, there cannot be good welfare, but good health alone does not guarantee good welfare (21). In the past, camels were thought to be resistant to diseases; however, this belief is no longer accurate (57). Currently, numerous viral, bacterial, and parasitic diseases affecting camels have been well-characterised (57–59). Diagnoses of these diseases are now frequently and accurately made in semi-intensive and intensive camel farming (60). However, most camel populations are managed under pastoralist nomadic environments, and in these nomadic pastoral communities, it is hard to adhere to animal health standards used in Western livestock systems (61). It is therefore important to enhance the veterinary health services also in those areas, to ensure the principle of good health.

“Female and male reproduction” (topic 5) was the fifth most important topic identified. It is critical to ensure sustained high levels of reproduction in camels, not only for profitable production but also to provide abundant possibilities for selection and genetic improvement (62). Despite an increase in camel production, in different Asian and African nations they are still managed under the traditional system records, making it difficult to implement genetic improvement (63). To exploit the full potential of camels, genetic improvement is essential and artificial insemination is highly needed (64). In fact, through artificial insemination, it is possible to prevent the spread of venereal diseases and allow the genes of highly valuable bulls to be spread more widely. However, the implementation of semen collection and artificial insemination is still problematic in Dromedary camels (65). Camels are seasonal breeders (66), and during breeding season males become restless and aggressive (22). To prevent aggressions, bulls are often individually stabled, and movement restriction, reduced space and lack of social contact can lead to stereotypical behaviours and impaired welfare (47, 67, 68). The collection of semen is done using either electro-ejaculation or an artificial vagina (AV) (65). The collection of semen utilizing electroejaculation is a welfare concern and not recommended since it requires the use of sedation or anaesthesia and is life-threatening, furthermore, the amount of sperm collected by this technique varies greatly (65). Currently, despite the large gap with other livestock species, efforts are being made to improve and make extensive usage of assisted reproductive technologies to improve the reproductive efficiency of camels, such as embryo transfer and artificial insemination (11). However, more work is needed to implement welfare-friendly reproduction techniques.

The limitations related to the method used to realise the present literature review must be reported. Firstly, synonyms of the words used in the search strings may have not been considered, leading to a reduction in the number of records that could have been included. Secondly, records not included in Scopus^®^ were not considered, and the same was for the “grey literature,” which is not included in Scopus. Furthermore, parameters of the search, such as the English-only language of the abstracts or specific subject areas, and the screening criteria adopted may have reduced the number of records analysed. Finally, the method of analysis used in the present review implied that the 234 records were not fully read but considered only from the title and abstract. Notwithstanding these limitations, this study reviewed the literature related to camel welfare, identifying the leading topics of camel scientific research and the gaps in knowledge about this animal species.

## 5. Conclusion

Through the utilization of text mining and topic analysis techniques, this review has identified and emphasised the most frequently investigated topics in Dromedary camel research related to animal welfare. Additionally, this study has shed light on the areas of camel welfare that remain unexplored and in need of further research. The result also indicates that there is exponential growth in the literature on Dromedary camel welfare. A higher number of records come from those countries where there is a growth of Dromedary camel populations and from traditional camel-rearing countries in Asia. The LDA identified the most important topics dealing with aspects of husbandry, management and welfare of Dromedary camels, milk production and calf management, behaviour and feeding management, camel welfare, camel health, and management system, and heading to female and male reproduction. Moreover, this review shows that although camel behaviour and welfare have received more attention recently from academia there is a need for more research to help improve our understanding of the welfare-related issues of Dromedary camels. Lastly, despite the limitations, this review gives an overview of the landscape of the camel welfare literature, highlighting both the most widely covered topics and those that still need in-depth study by scientists around the world.

## Author contributions

NTM: Data curation, Investigation, Writing – original draft. MZ: Conceptualization, Investigation, Methodology, Writing – original draft. MF: Conceptualization, Data curation, Investigation, Methodology, Writing – review & editing. BB: Conceptualization, Investigation, Methodology, Resources, Writing – original draft. BP: Conceptualization, Funding acquisition, Investigation, Methodology, Project administration, Supervision, Writing – review & editing.

## Footnotes

^1^ www.wordclouds.com

^2^ www.icc-gic.weebly.com
