# Supplementary material for: Dromedary camel’s welfare: literature from 1980 to 2023 with a text mining and topic analysis approach
Source: Front Vet Sci. 2023 Nov 9;10:1277512. doi: 10.3389/fvets.2023.1277512 (PMC10665734; doi:10.3389/fvets.2023.1277512)
Supplement: Supplementary file 1 [file Data_Sheet_1.pdf]

Supplementary Material

Dromedary camel's welfare: literature from 1980 to 2023 with a text mining approach

Naod T Masebo, Martina Zappaterra, Martina Felici*, Beatrice Benedetti, Barbara Padalino

*** Correspondence:** Martina Felici: martina.felici6@unibo.it

1 Supplementary Figures and Tables

1.1 Supplementary Figures

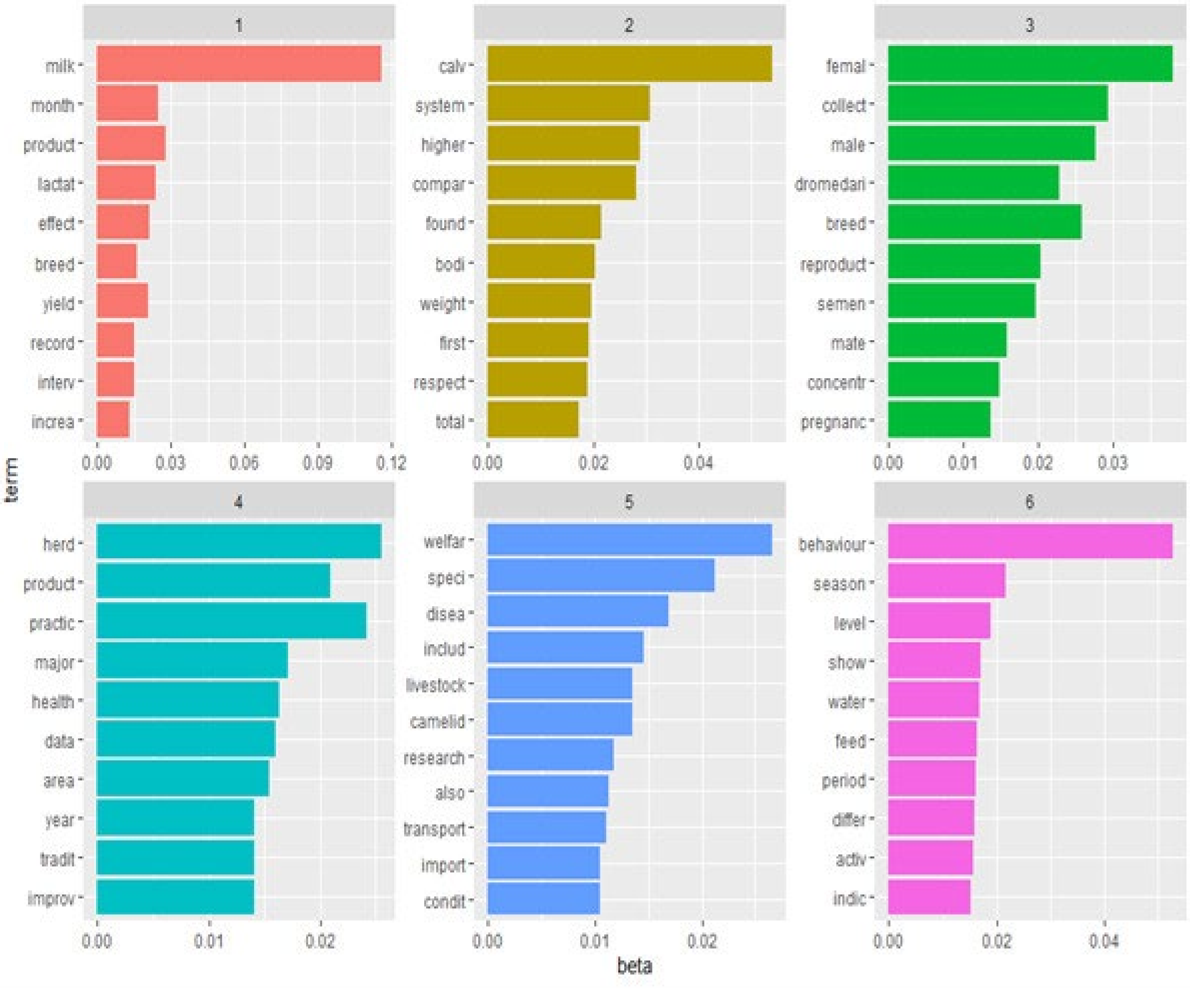

Supplementary Figure 1. The TA performed with 6 a-priori numbers of topics.

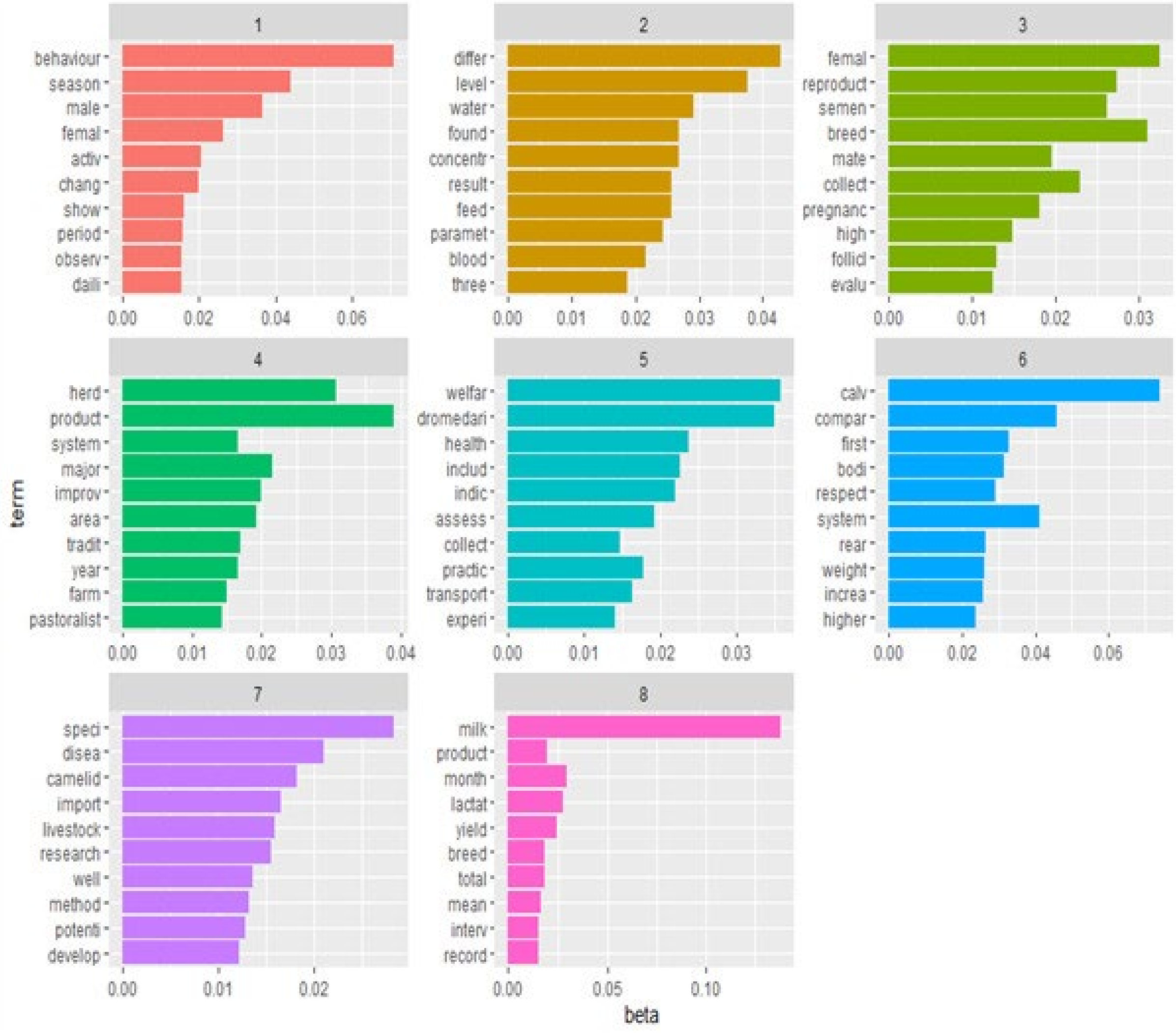

Supplementary Figure 2. The TA performed with 8 a-priori numbers of topics.
